# Mesenchymal Stromal Cell Secretome Is Affected by Tissue Source and Donor Age

**DOI:** 10.1093/stmcls/sxad060

**Published:** 2023-08-17

**Authors:** Agnieszka J Turlo, Dean E Hammond, Kerry A Ramsbottom, Jamie Soul, Alexandra Gillen, Kieran McDonald, Mandy J Peffers

**Affiliations:** Department of Musculoskeletal and Ageing Science, Institute of Life Course and Medical Sciences, University of Liverpool, Liverpool, UK; epartment of Cellular and Molecular Physiology, Institute of Systems, Molecular and Integrative Biology, University of Liverpool, Liverpool, UK; Computational Biology Facility, Liverpool Shared Research Facilities, Faculty of Health and Life Sciences, University of Liverpool, Liverpool, UK; Computational Biology Facility, Liverpool Shared Research Facilities, Faculty of Health and Life Sciences, University of Liverpool, Liverpool, UK; Department of Veterinary Science, Philip Leverhulme Equine Hospital, University of Liverpool, UK; Biobest Laboratories Ltd, Liverpool, UK; Department of Musculoskeletal and Ageing Science, Institute of Life Course and Medical Sciences, University of Liverpool, Liverpool, UK

**Keywords:** mesenchymal stromal cells, secretome, proteomics, age, equine, donor

## Abstract

Variation in mesenchymal stromal cell (MSC) function depending on their origin is problematic, as it may confound clinical outcomes of MSC therapy. Current evidence suggests that the therapeutic benefits of MSCs are attributed to secretion of biologically active factors (secretome). However, the effect of donor characteristics on the MSC secretome remains largely unknown. Here, we examined the influence of donor age, sex, and tissue source, on the protein profile of the equine MSC secretome. We used dynamic metabolic labeling with stable isotopes combined with liquid chromatography-tandem mass spectrometry (LC-MS/MS) to identify secreted proteins in MSC conditioned media (CM). Seventy proteins were classified as classically secreted based on the rate of label incorporation into newly synthesized proteins released into the extracellular space. Next, we analyzed CM of bone marrow- (*n* = 14) and adipose-derived MSCs (*n* = 16) with label-free LC-MS/MS. Clustering analysis of 314 proteins detected across all samples identified tissue source as the main factor driving variability in MSC CM proteomes. Linear modelling applied to the subset of 70 secreted proteins identified tissue-related difference in the abundance of 23 proteins. There was an age-related decrease in the abundance of CTHRC1 and LOX, further validated with orthogonal techniques. Due to the lack of flow cytometry characterization of MSC surface markers, the analysis could not account for the potential effect of cell population heterogeneity. This study provides evidence that tissue source and donor age contribute to differences in the protein composition of MSC secretomes which may influence the effects of MSC therapy.

## Graphical Abstract

**Figure:**
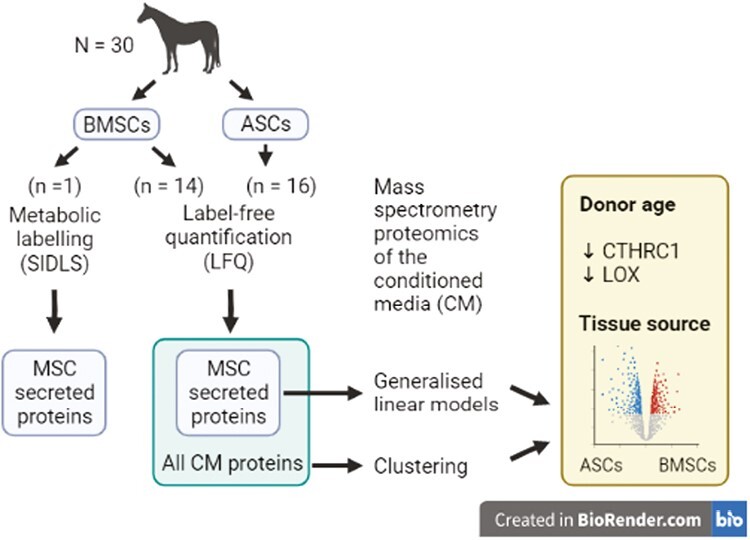

**Significance Statement**

This research shows for the first time that donor age can influence the proteins secreted by mesenchymal stromal cells (MSC), which is considered the main mechanism through which they mediate their biological actions. This information may improve our understanding of the variable outcomes observed in MSC clinical studies, as well as identify optimal sources and donor selection for specific clinical applications.

## Introduction

Mesenchymal stromal cells (MSCs) are widely investigated as regenerative therapy in human and veterinary medicine, based on their capacity for tissue remodeling and immunomodulation. However, translation of MSCs into clinical practice has been problematic due to heterogeneity of MSC populations^1^ that is influenced, among others, by donor characteristics.^2,3^ Advancing knowledge of the donor-related effect could improve standardization of MSC therapy through purposeful selection of cell sources and identify cases when an allogenic approach may be beneficial.

Ageing leads to a decline in function of all cell types, making donor age one of the main considerations in studying MSC variability. MSC therapy is often used in an autologous manner to address potential ethical and safety concerns related to the use of allogenic cell sources.^2,3^ Age is also a risk factor for diseases investigated as targets for MSC therapy such as osteoarthritis,^4^ tendinopathy,^5^ neurodegeneration,^6^ or macular degeneration.^7^ Consequently, MSCs applied in a clinical setting are likely to be isolated from older patients, highlighting the importance of understanding the effect of age on MSC potency.

Donor age and sex affect multiple MSC functions including proliferation and differentiation,^8-15^ immunomodulation,^16^ gene expression,^14,17-19^ DNA methylation,^20,21^ and energy metabolism.^20^ However, the effect of donor characteristics on protein secretion in MSCs is less well explored. Secreted factors are the main mechanism mediating MSC action and have been investigated as a potential cell-free therapy.^2,22^ One of the key features of cellular ageing is the senescence-associated secretory phenotype (SASP) that can induce age-related pathologies in surrounding tissue. SASP markers derived from in vitro ageing studies of fibroblasts and epithelial cells^23^ were used to test the effect of ageing on MSC protein secretion.^24-28^ However, as SASP components vary across cell types,^29^ comprehensive proteomic analysis is required to characterize the age-related changes in MSC protein secretion.

Untargeted proteomics was previously used to investigate differences in composition of conditioned media (CM) from MSCs from various tissue sources^30-33^ and different passages of a long-term cell culture, serving as an in vitro model of ageing.^33-37^ In vitro ageing does not reflect the complexity of chronological ageing, and these two processes were previously shown to have a different impact on the function of rat adipose-derived MSCs.^38^ Therefore, MSC data from donors of different ages are needed to fully assess the relationship between MSC ageing and secreted proteins.

This study aimed to test the effect of donor sex, age, and tissue source on the proteins secreted by equine MSCs using targeted and untargeted proteomics methods. The results demonstrate that donor age and sex are not likely to drive global changes in MSC CM proteome; however, donor age affects the abundance of individual proteins that may be relevant to tissue repair.

## Materials and Methods

### Cell Collection and Culture

The owners of the horses included in the study have been made aware that tissues may be retained for research purposes and provided informed consent for their use in research. Bone marrow-derived MSCs (BMSCs) were obtained for the purpose of autologous cell therapy and provided by Biobest Laboratories Ltd. Surplus samples of BMSCs from treatment were cryopreserved and stored for research use. Equine adipose-derived MSCs (ASCs) were isolated from retroperitoneal adipose tissue obtained as a waste tissue from colic (abdominal) surgery, where it was necessary to remove small quantity to aid closure of laparotomy incision. Tissue collection for research was approved by the Committee on Research Ethics, University of Liverpool School of Veterinary Science (RETH000689).

All tissue culture reagents were sourced from Gibco. Adipose tissue was placed in cold phosphate buffer saline (PBS) with 1% penicillin/streptomycin. ASCs were isolated following tissue digestions with collagenase type I as previously described^39^ (Supplementary Material 1) and cultured in an expansion culture medium, consisting of low-glucose Dulbecco’s modified Eagle’s medium (DMEM), supplemented with 30% fetal bovine serum (FBS), 100 U/mL penicillin/streptomycin, and 2.5 µg/mL of amphotericin B (expansion medium). After reaching 70% confluence, ASCs were passaged and cryopreserved in liquid nitrogen.

BMSC and ASC samples were thawed, seeded in T75 flask and cultured in expansion medium up to 70% confluence before passaging for use in cell characterization experiments and generation of CM. In all experiments, cells were used at passage 2 or 3, with medium changed twice a week, unless stated otherwise. The cells were cultured at 37 °C in humidified atmosphere with 5% CO_2_ and 5% O_2_. Hypoxic conditions were shown to enhance paracrine therapeutic potential of MSCs through increased secretion of trophic factors^40^; moreover, they imitate the environment of the structures most commonly treated with MSCs in the horse (tendon and joint) which makes them a relevant model for our study.

### Tri-lineage Differentiation Assay

MSC samples were assessed for their ability to differentiate into osteoblasts, adipocytes, and chondrocytes following induction with commercial differentiation media (StemPro, Gibco).^41^ Adipogenic and osteogenic differentiation were undertaken in a monolayer culture while chondrogenic differentiation was tested in pellet culture (Supplementary Material 1).^42^ Cells cultured in expansion medium were used as a negative control and results assessed through specific stains (Sigma–Aldrich); Alizarin Red for osteogenic, Oil Red O for adipogenic and Alcian Blue for chondrogenic differentiation.

Microscopic images of pellet cultures were taken before processing for histology, to measure their size using ImageJ.^43^ The area of the cell pellet and the field of view (FOV) were measured, and pellet size reported as a fraction of FOV. The difference between the size of chondrogenic and control pellet for each tissue type was analyzed with a paired *t*-test and means considered significantly different when related *P*-value was<.05.

### Expression of MSC Markers

RNA from MSC samples was extracted with TRIzol reagent (Invitrogen) and RNeasy Mini Kit (Qiagen) following manufacturers protocols. Reverse transcription was performed using 1 µg RNA and 0.5 µg random primers, 200U of M-MLV reverse transcriptase, 25U of RNAsin ribonuclease inhibitor and 500 µM dNTP deoxynucleotide mix (all reagents from Promega). Gene expression of CD90, CD29, CD44, CD105, CD34, CD45, CD79α, and glyceraldehyde-3-phosphate dehydrogenase (GAPDH) was measured by RT-qPCR using Takyon SYBR Master Mix (Eurogentec), 100 nM of specific primers, using previously published sequences^44^ (Table 1), and Roche Lightcycler 480 (Roche). Expression of MSC marker genes relative to GAPDH was calculated using the comparative Ct method.^45^ Sample without the RNA template served as a negative control.

**Table 1.** Primer sequences used for equine MSC characterization.

Gene symbol	Forward primer 5ʹ-3ʹ	Reverse primer 5ʹ-3ʹ
CD90	TGCGAACTCCGCCTCTCT	GCTTATGCCCTCGCACTTG
CD29	GTGAGATGTGTCAGACGTGC	AGAACCAGCAGTCATCCACA
CD44	TTCATAGAAGGGCACGTGGT	GCCTTTCTTGGTGTAGCGAG
CD105	GACGGAAAATGTGGTCAGTAATGA	GCGAGAGGCTCTCCGTGTT
CD34	CTCCAGCTGTGAGGACTTTA	AAGTTCTGGATCCCCATCCT
CD45	CTCCTCATTCACTGCAGAGA	GGTACTGCTCAAATGTGGGA
CD79α	AGGGAGCCACATCAACATCA	CGTTGCCTTCCTTAGCTTGG
GAPDH	GGGTGGAGCCAAAAGGGTCATCAT	AGCTTTCTCCAGGCGGCAGGTCAG

### Stable Isotope Dynamic Labeling of Secretomes

To distinguish proteins secreted by MSCs from the structural proteins released into culture media through cell damage, we used dynamic labeling with stable isotopes.^46^ This method used the incorporation of stable isotope-labelled amino acids into newly made proteins released from cells over time, to infer their rate of synthesis and subsequent secretion. For this experiment, one sample of BMSCs at passage 4 was seeded in 4 T25 flasks at 5000 cells/cm^2^. At 80%-90% confluence, expansion media was removed, cells washed 3 times with PBS, and 5ml of serum-free DMEM deficient in lysine and arginine (Gibco) with (13C6)-labelled l-lysine (CK Isotopes Ltd, UK) and (13C6/15N4)-labelled arginine (CK Isotopes Ltd, UK) was added to each flask. CM was collected after 1 hour, 2 hours, 6 hours, or 24 hours incubation (one flask per time point), centrifuged at 800*g* for 7 minutes to remove cell debris, flash frozen, and stored at −80°C prior to mass spectrometry analysis.

### Label-Free CM Collection

Label-free CM were obtained from all MSC samples to investigate the association between protein abundance and donor age, sex and tissue source. Each MSC sample was seeded into 2 T25 flasks at 5000 cells/cm^2^. CM from one of the replicates was used for mass spectrometry analysis while the other for validation of the results with orthogonal techniques. At 80%-90% confluence, expansion media was removed, cells washed 3 times in PBS and 5 mL of serum-free expansion media added. After 24 hours of incubation media samples were collected, centrifuged at 800*g* for 7 minutes to remove cell debris, flash frozen, and stored at −80°C.

### Protein Concentration with StrataClean

Isotope labelled and label-free media were analyzed in two separate mass spectrometry experiments. Protein concentration in CM samples was measured with Pierce 660 nm assay (Thermo Fisher Scientific) following the manufacturer’s protocol. Protein was concentrated using the StrataClean resin (Agilent Technologies).^46^ CM volume for StrataClean binding was adjusted to obtain 100 µg of total protein. In samples with lower protein concentrations, the whole volume of 5 mL CM was used. Each CM sample was mixed with 12 µL of StrataClean resin, vortexed for 1 minute, then mixed by inverting for 10 minutes. Samples were centrifuged at 295*g* for 1 minute and protein-depleted supernatants discarded. StrataClean resin was washed with LC-MS grade water (Thermo Fisher Scientific) and centrifuged at 295*g* for 1 minute, twice, followed by on-bead protein digestion.^46^

### Protein Digestion for LC-MS/MS

The on-bead protein digestion with trypsin and lysyl endopeptidase (LysC) was performed as described previously.^46^ Details of experimental method are included in Supplementary Material 1.

### LC-MS/MS Spectral Acquisition

Mass spectrometry analysis of the secretome samples was undertaken in the Centre for Proteome Research at the University of Liverpool. Protein digest samples were analyzed using an Ultimate 3000 RSLC nano-system (Thermo Fisher Scientific) coupled to a Q Exactive HF Quadrupole-Orbitrap mass spectrometer (Thermo Fisher Scientific) and a 2-hour gradient. Details of analytical method are included in Supplementary Material 1.

### Protein Identification and Quantification

Mass spectrometry data from two experiments were analyzed separately using MaxQuant software (v1.6.17.0 [Stable Isotope Dynamic Labeling of Secretomes, SIDLS] and v2.0.3.0 [label-free], Max Planck Institute of Biochemistry).^47-49^ Proteins were identified using UniProt reference proteome for *E. caballus* (9796, accessed May 11, 2021 and May 10, 2022, 20 861 entries) and Andromeda search engine.^49^ MaxQuant default search parameters were used (Supplementary Material 1). Peptide and protein false discovery rate (FDR) were set to 1% for reporting of peptide spectrum matches (PSM) and proteins.

In addition, in analysis of SIDLS data, labels Lys8 and Arg10 were selected to enable identification of isotope-labeled peptides. In analysis of label-free data, label-free quantification (LFQ), a MaxQuant intensity determination and normalization algorithm, was enabled.^47^

### Kinetic Analysis of Secreted Proteins Identified With SIDLS

Data from SIDLS experiment were analyzed following the previously published method^46^ using the R (v4.1.0) programming language environment. MaxQuant “evidence.txt” search results file was used as input data. First, reverse and contaminant peptides were removed. For each identified peptide, at each time point, the relative isotope abundance (RIA) was calculated by dividing abundance of the isotope-labeled (heavy) peptide by summed abundance of labeled and non-labeled (light) peptide (RIA = H/[H + L]). To model protein labeling over time (RIA trajectory), we used only peptides identified and quantified in at least 3 time points. To obtain protein-level kinetic data, we grouped RIA data for peptides assigned to the same protein (“Leading razor protein”). For each peptide subset, changes of RIA over time were modeled as a first-order rate process and fitted via non-linear least squares as described previously.^46^ Next, we calculated the difference in the total mean abundance of peptides (H + L), for each protein, between 24- and 6-hour time point (P) and used this to calculate protein flux from the cell to extracellular space, by multiplying this with the first-order rate constant (*k*) at which each protein acquired isotopic label. To differentiate secreted from intracellular proteins, we plotted the rate of label incorporation (*k*) against log10 flux for each protein and empirically selected a cutoff value of *k* to separate and classify proteins with distinct kinetic properties.

### Validation of Kinetic Protein Classification

To validate protein classification based on *k* and their flux through the protein pool, we cross-annotated the protein list with UniProt Gene Ontology (GO) terms for cellular component (CC) and undertook GO enrichment analysis of the secreted and intracellular protein subsets using the R package clusterProfiler (v4.2.2).^50,51^ The list of all proteins identified across all 4 time points (271) was used as a background for over-representation analyses. Gene symbols were used as an input and searched against human gene annotation database (“org.Hs.eg.db”) due to lack of species-specific database supported by R.

### Clustering Analysis of Label-Free Mass Spectrometry Data

For label-free dataset analysis, MaxQuant output file “proteinGroups.txt” was used. Reverse and contaminant hits were removed, and only proteins with 2 unique peptides retained for statistical analysis.

First, we investigated the missing protein abundance values. In order to allow for a reliable analysis using a more complete dataset, we removed proteins that showed more than 30% missing values across all samples. To investigate the impact of missing values on our analysis, we compared the effects of using different imputation methods on clustering analysis; (i) no imputation, (ii) sample-based imputation and, (iii) protein-based imputation. A detailed explanation of these methods is included in the Supplementary Material 1.

We have undertaken an unsupervised overview of the data using principal component analysis (PCA) to compare the effects of tissue type and sex. We then analyzed each tissue separately and clustered the samples based on age. As the ages of the samples were variable and few samples shared ages with other samples, it was decided to group the samples in age range bins of 5-year intervals. This allowed identification of clustering patterns associated with age as each group consisted of at least 3 samples.

Next, we used time course clustering to purposefully identify co-expressed genes across different ages. We used 2 similar methods; dynamic time clustering (R package DTWclust v1.23-1)^52^ and fuzzy time course clustering (Mfuzz v2.56.0).^53^ We first took the mean protein abundance for duplicated samples (ie, samples with the same tissue type, sex, and age). Average values per sample were then standardized at a protein level and the samples ordered by age. For DTWclust, 8 clusters were used, and the proteins assigned to each cluster identified. Mfuzz was used in a similar way, this time specifying 16 clusters. From the time course clustering approaches, it was hoped that we aimed to identify trends in protein expression over time (ie, age) for each of the assigned clusters.

Finally, another unsupervised clustering method was used to identify what drove the variation in protein abundance within tissues. This was completed with non-negative matrix factorization (NMF), using the R package NMF (v0.25).^54^ In order to choose the cluster number, we used non-smooth NMF testing between 2 and 6 clusters, in order to evaluate the stability of the cluster allocations. We chose to complete the NMF using 4 clusters, as this showed the most stable results, and visualized the association of clusters with each of the known variables (donor age, sex, and tissue source).

### Generalized Linear Models of Protein Abundance

To investigate associations between the abundance of secreted proteins and donor age, sex, and tissue type, a generalized linear model (GLM) was applied to each protein identified as secreted using SIDLS and also identified in the label-free dataset obtained from 30 independent MSC samples. The observed protein abundance was modeled as a Gamma distribution to reflect the positive-only and skewed data. The effect of predictors was considered significant when *t*-statistics correspond to *P* < .05, following adjustment for multiple testing with Benjamini-Hochberg method (*q* < 0.05). Samples with missing data were excluded from analysis on an individual protein basis and information regarding the sample size used in each model reported. For proteins where a categorical predictor (sex, tissue type) was considered to have a significant effect on abundance, the log2-fold change in abundance between conditions was calculated.

### Lysyl Oxidase Activity Assay

The activity of lysyl oxidase (LOX) in CM samples (*n* = 27) was measured using fluorometric commercial assay (Abcam, ab112139) following the manufacturer’s protocol. The relationship between LOX activity and donor age was evaluated using generalized linear mixed-effects models with incubation time, age, and a time-by-age interaction term as fixed effects, individual donor as a random effect and normalized fluorescence as the dependent variable (Supplementary Material 1).

### Western Blot Analysis of Collagen Triple Helix Repeat-Containing Protein 1

CM samples with the highest and lowest Collagen Triple Helix Repeat-Containing Protein 1 (CTHRC1) abundance, according to the LC-MS/MS analyses, from each tissue source, were selected for western blotting (*n* = 6). Samples were concentrated using StrataClean resin as described previously,^55^ subjected to sodium dodecyl-sulfate polyacrylamide gel electrophoresis and stained with rabbit anti-human CTHRC1 antibody (Abcam, ab85739, Supplementary Material 1). Fibronectin detected using an anti-human antibody (Sigma-Aldrich, F3648) was used as a loading control.^56^ To quantify the CTHRC1 abundance, the relative density of CTHRC1 band (ratio of CTHRC1 to FBN) was evaluated using ImageJ (Supplementary Material 1).^43^

### ELISA Analysis of Monocyte Chemoattractant Protein 1

There was insufficient information regarding the abundance of a dominant marker of SASP, Monocyte Chemoattractant Protein 1 (MCP-1), in the mass spectrometry dataset (>30% missing values). Therefore, we have used a commercial sandwich enzyme-linked immunosorbent assay (Abcam, ab272035) to measure concentration of MCP-1 in MSC CM (*n* = 24) following manufacturer’ protocol. The relationship between donor profile and tissue source and MCP-1 concentration was tested using GLMs as described for the mass spectrometry data (Supplementary Material 1).

## Results

### Characterization of Equine MSCs

**Figure 1.**
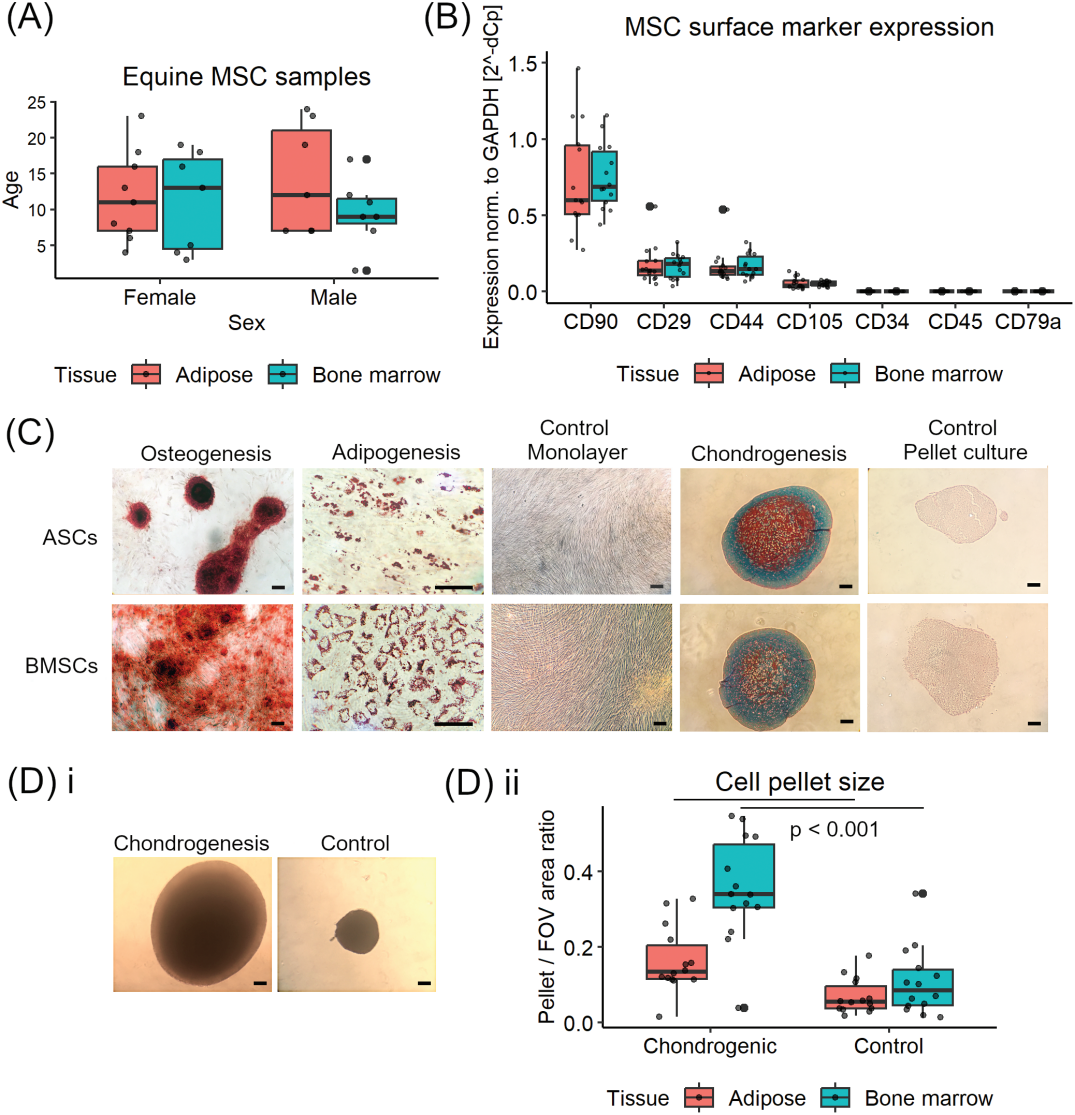
Characterization of equine mesenchymal stromal cells (MSC). (**A**) Distribution of MSC samples (*N* = 30) with regard to donor age, sex, and tissue type used for cell isolation. (**B**) Expression of MSC surface marker genes relative to glyceraldehyde-3-phosphate dehydrogenase (GAPDH), measured with qRT-PCR. (**C**) Microscopic images of adipose- and bone marrow-derived MSCs grown as monolayers in osteogenic induction media and stained with Alizarin Red; adipogenic induction media and stained with Oil Red O; grown in pellet culture in chondrogenic induction media, paraffin sectioned and stained with Alcian Blue. Control cultures were grown in MSC expansion media. Representative images from 6 biological replicates. Bar: 100 μm. (**D**) Representative microscopic images (*n* = 1, bone marrow-derived MSCs) used for MSC pellet size measurements (i) and graph showing relative size of pellets grown in chondrogenic and control media (ii). Bar: 100 μm. Different letters indicate statistically significant (*P* < .05) differences between chondrogenic and control group in samples from the same tissue source, determined with paired *t*-test (ASC, *n* = 14; BMSC, *n* = 14). In all box plots, horizontal lines represent median, boxes interquartile range, whiskers range, and dots biological replicates.

This study used equine MSCs isolated from bone marrow (*n* = 14) and adipose tissue (*n* = 16) collected from horses aged from 1.5 to 24 years (mean 11.7 years), 14 males and 16 females (Fig. 1A). MSCs from both tissue sources showed positive relative expression of genes encoding MSC surface marker proteins CD90 (mean = 0.79), CD29 (mean = 0.17), CD44 (mean = 0.17), and CD105 (mean = 0.05), and low expression of CD34, CD45 and CD79α (mean < 0.001), indicating that the cell populations used did not include hematopoietic stem cells or B cells (Fig. 1B). Moreover, CD44 and CD29 proteins have been identified in the label-free mass spectrometry analysis of equine MSC CM. Surface proteins in CM were likely derived from extracellular vesicles secreted by MSCs, which have been shown to carry the same surface protein signature.^57,58^ Staining of MSC cultures with Alizarin Red following osteogenic induction showed the presence of calcium deposits and staining with Oil Red O showed the presence of lipid droplets in adipogenic cultures (Fig. 1C). Paraffin sections of chondrogenic MSC pellets showed positive staining for Alcian Blue, indicating presence of glycosaminoglycans (Fig. 1C). The mean size of MSC pellet cultures maintained in chondrogenic media was 2.5- (ASC) and 3.5- (BMSC) times higher than control pellets (*P* < .05, in both tissue types, Fig. 1D).

### Stable Isotope Labeling Identified Secreted Proteins in MSC CM Based on Their Kinetic Behavior

**Figure 2.**
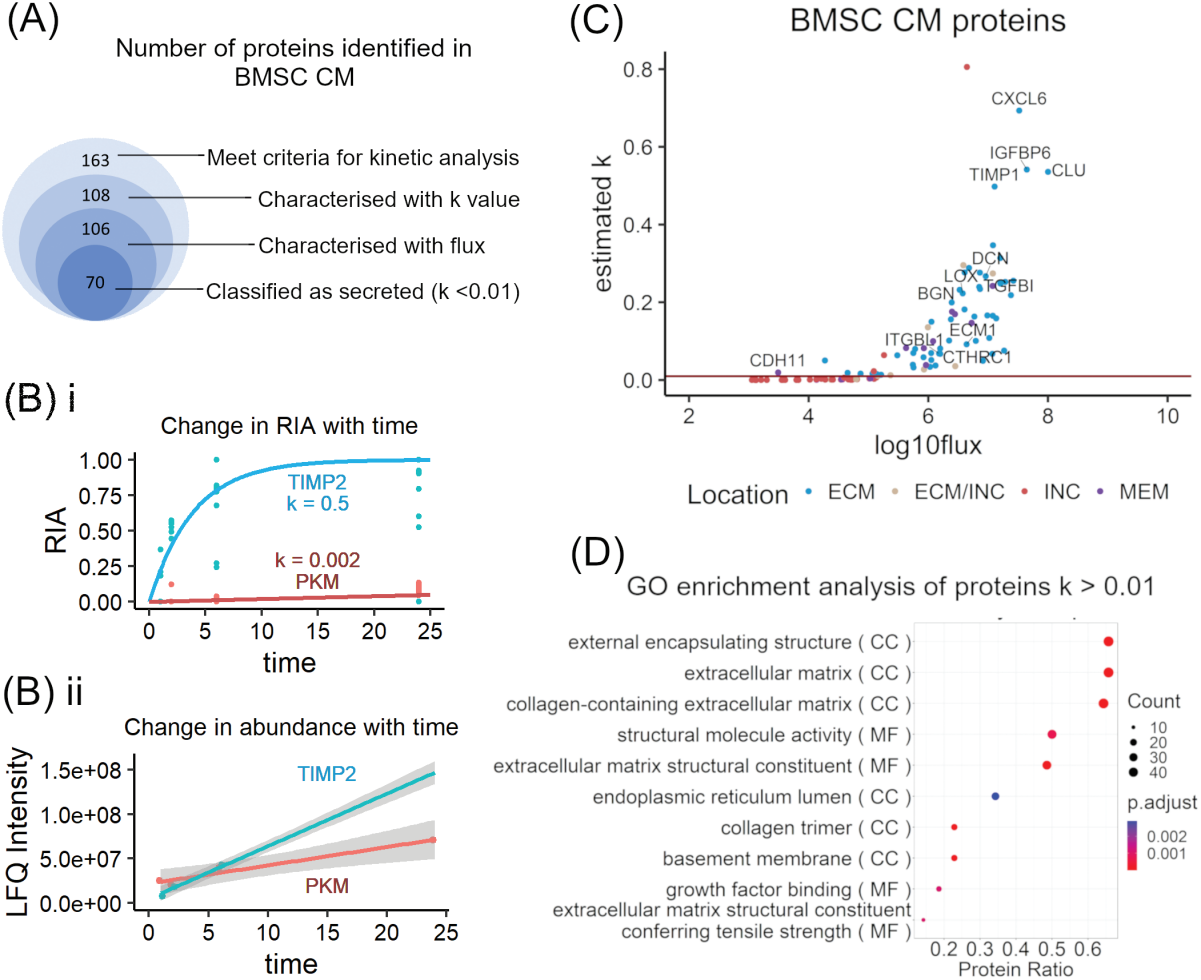
Stable Isotope Dynamic Labeling of Secretomes (SIDLS) allowed identification of classically secreted proteins in conditioned medium (CM) of equine Mesenchymal Stromal Cells (MSCs). (**A**) Stacked Venn diagram showing number of proteins at consecutive stages of mass spectrometry data analysis (identified in at least 3 out of 4 time points, *n* = 1). The innermost circle shows the number of proteins characterized as secreted, based on their kinetic properties. (**B**) Dynamic behavior of representative secreted (metalloproteinase inhibitor 1, TIMP1) and intracellular (pyruvate kinase, PKM) proteins. The relative isotope abundance (RIA) describes the ratio of heavy-labelled peptide abundance to the sum of labelled and non-labelled peptide abundances. TIMP1 exists in a small intracellular pool, acquires heavy-labelled amino acid quickly during nascent de novo protein synthesis, while the intracellular pool of PKM is large and acquires heavy labels over much longer time periods. Dots represent RIA values of single peptides and the lines the fitted curves for a first-order equation (i). Total abundance of secreted proteins in CM increases in time faster than intracellular proteins derived from cell leakage/death. Dots represent mean protein abundance with regression lines and 95% CI (shaded area) (ii). (**C**) Scatterplot showing 106 proteins identified in MSC CM and characterized with kinetic parameters: first-order rate constant (*k*), describing change of Relative Isotope Abundance in time, and flux, describing the rate at which the protein flows from the intracellular to the extracellular space. Red line represents empirically selected threshold (*k* = 0.01) discriminating proteins with distinct kinetic behavior. Color-coding was based on gene ontology (GO) cellular component (CC) terms. Labels designate proteins with the highest secretion rate and those that were influenced by donor characteristics in this study. (**D**) Dot plot of GO term enrichment analysis of 70 proteins classified as secreted based on kinetic properties. The size of the dots indicates the number of proteins mapped to that term. The x-axis shows number of proteins that map to the term divided by the total number of secreted proteins. The dots are color coded by adjusted *P*-values (BH method).

Out of 163 proteins with peptides identified in CM samples in at least 3 of 4 time points, the rate of synthesis and secretion (*k*), and flux, for 106 proteins were characterized in detail (Fig. 2A, Supplementary Material 1). Based on an empirically selected cutoff value of *k* = 0.01, 70 proteins were classified as secreted and 36 as intracellular (Fig. 2B). Differences in dynamic behavior between secreted and intracellular proteins underlying this classification are illustrated in Fig. 2C.

Cross-annotation with GO cellular component terms showed that, out of 70 classified as secreted, 58 were confirmed as extracellular proteins, 5 of intracellular origin, 1 a membrane protein, and 6 were assigned to multiple (intra- and extracellular) locations. All proteins with *k* < 0.01 were assigned to the intracellular pool or multiple locations (Fig. 2B). As expected, GO enrichment analysis of proteins with *k* > 0.01 resulted in top cellular component and molecular function terms associated predominantly with extracellular matrix (ECM) structure (Fig. 2D). The full list of 106 proteins and their kinetic parameters are available in Supplementary Material 2.

### Tissue Origin Is the Main Factor Affecting Protein Composition of Equine MSC CM

Label-free quantification analysis of equine MSC CM samples from 30 donors identified 1164 proteins based on the presence of minimum 2 unique peptides. Following filtering for proteins where quantification values were missing in over 30% of samples, 716 proteins were used for clustering analysis using non-imputed, sample-imputed, and protein-imputed datasets, which showed that imputation did not affect the sample clustering patterns (Supplementary Material 1; Supplementary Fig. S1). Therefore, we decided to present results of the analysis using only the non-imputed variables (314 proteins).

**Figure 3.**
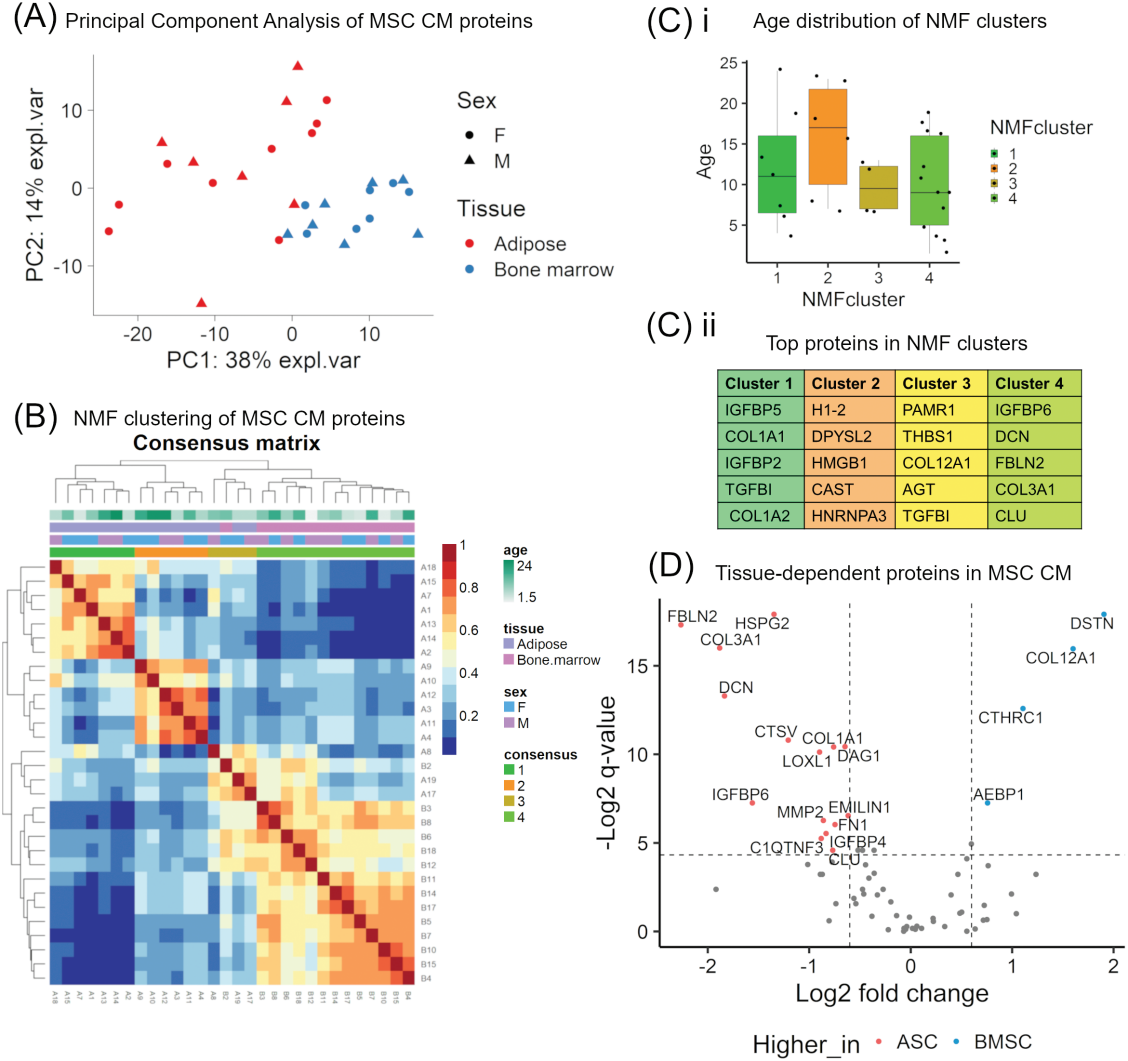
Tissue source is the main factor associated with differences in the abundance of secreted proteins in equine mesenchymal stromal cell (MSC) conditioned media (CM). (**A**) Principal component analysis (PCA) biplot of label-free mass spectrometry data obtained from MSC CM samples (*n* = 30) showing the first 2 principal components. Each dot represents a biological replicate with color denoting the tissue type used for MSC isolation and shape donor sex. (**B**) Consensus matrix from non-negative matrix factorization (NMF) clustering of MSC CM samples based on the normalized abundance values (LFQ intensity) of 314 proteins identified in all biological replicates with mass spectrometry. Four distinct clusters were identified (color-coded as “consensus”) and mapped against MSC source characteristics (color-coded age, tissue, and sex). (**C**) (i) Boxplots showing distribution of donor age in samples allocated to 4 NMF clusters; (ii) gene names of the top proteins identified in each NMF cluster (color code consistent with consensus matrix). Abundance of those proteins was the main source of similarity between MSC CM samples allocated to each cluster. (**D**) Volcano plot showing 69 proteins classified as secreted by SIDLS that were further identified with label-free mass spectrometry in MSC CM samples (*n* = 30). The x-axis shows log2-fold change in mean abundance of each protein (dots) in bone marrow- relative to adipose-derived MSC CM. The y-axis shows the −log2 *q*-value (FDR adjusted *P*-value, BH method) associated with the effect of tissue source on protein abundance as determined with a generalized linear model (GLM). Proteins with log2-fold change >.6 or <−0.6 and *P*-value <.05 were color-coded and labelled with gene names.

PCA showed that tissue origin of MSCs is the main variable driving clustering of CM samples, accounting for up to 52% of variance in protein abundance (Fig. 3A). There was no clear association between donor sex or age and sample clusters, including after removing the tissue factor (Fig. 3A, Supplementary Fig. S2A). The unsupervised clustering using NMF method resulted in 4 distinct sample clusters. The number of clusters was assigned based on the NMF clustering rank validation (Supplementary Fig. S2B). One of the clusters (cluster 4) included 13 out of 14 BMSC CM samples, while ASC CM samples were grouped into 3 subclusters (Fig. 3B), supporting the finding that tissue type is driving similarity in protein abundance across MSC CM samples. NMF clusters did not appear to be associated with sex or age of the donor (Fig. 3Ci). Top proteins driving similarity between samples within each cluster included mainly extracellular (secreted) proteins, apart from the cluster 2, that was characterized by cytosolic and membrane proteins (Fig. 3Cii). The full list of protein ranks is available in Supplementary Material 3.

Out of 70 proteins classified as secreted by SIDLS, 69 were identified in label-free dataset and were progressed to the association study using GLMs described eariler. The null hypothesis of no effect of tissue origin could be rejected at the level of 0.05 for 23 proteins (Supplementary Material 4), and for 19 of these proteins the log2-fold change in mean protein abundance between tissue groups was larger than 1.5 (log2-fold change > 0.6 or < −0.6, Fig. 3D). Following adjustment for multiple comparisons (*q*-values) and log2-fold change, when applying a significance threshold of 0.05, 15 proteins were considered upregulated in ASC CM, while 4 were upregulated in BMSC CM (Fig. 3D). All of the proteins previously identified as most characteristic of BMSC CM cluster in NMF analysis were significantly downregulated in BMSC CM according to GLM and fold change analysis.

### Abundance of Proteins Related to ECM Remodeling Declines With Donor Age in Equine MSC CM

**Figure 4.**
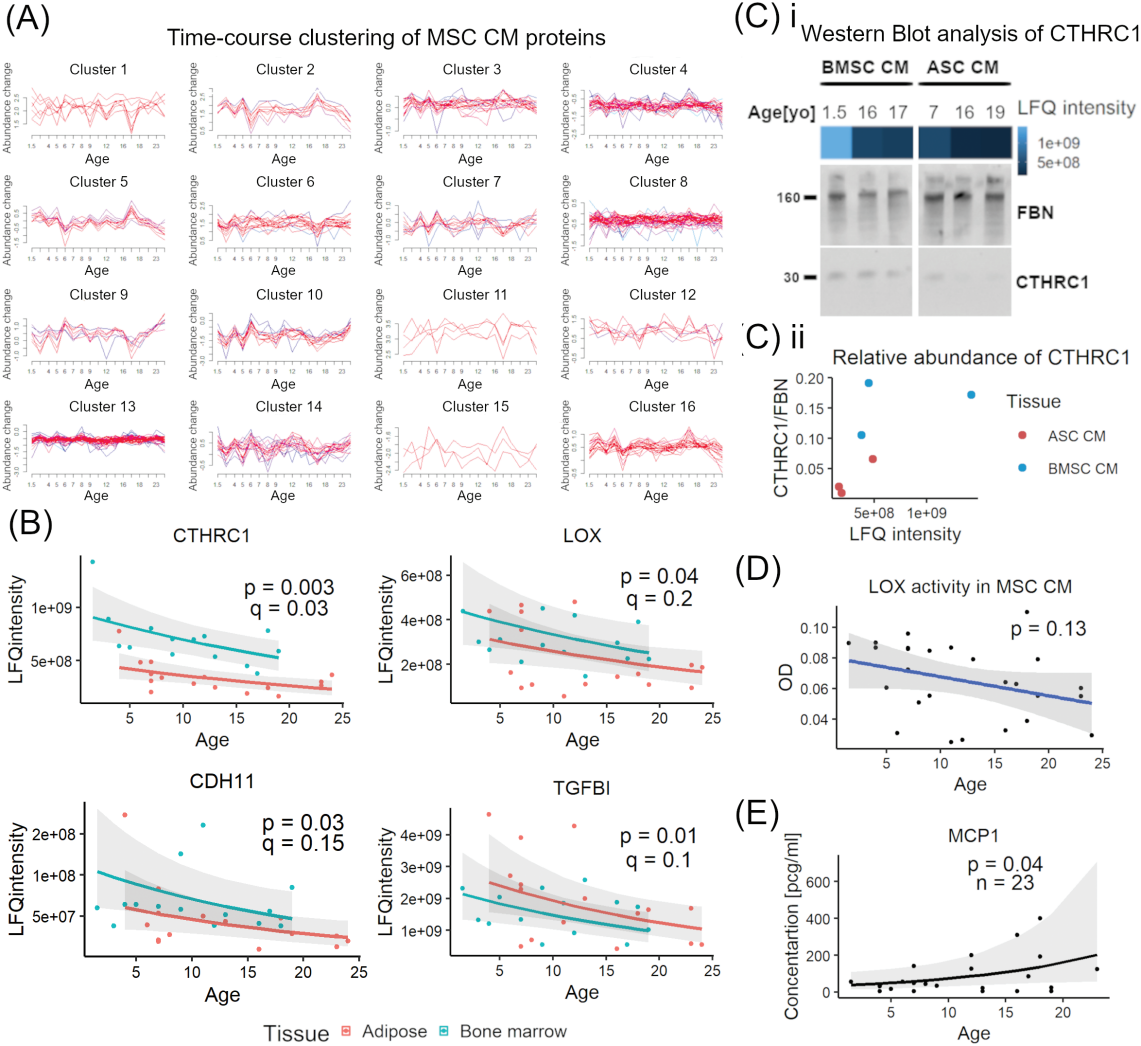
Donor age affects the abundance of selected proteins identified in equine mesenchymal stromal cell (MSC) conditioned media (CM). (**A**) Cluster members identified with fuzzy time course clustering. Graphs show clusters of proteins with similar abundance patterns and change in their abundance value with donor age. (**B**) Scatterplots showing change in CM protein abundance, measured by quantitative mass spectrometry, with MSC donor age. Dots represent biological replicates, colors tissue type used for MSC isolation, lines the mean protein abundance predicted by generalized linear model (GLM) and shades 95% CI. Proteins with the strongest relationship between age and abundance (*P*-value <.05, *q*-value <.2) are shown: collagen triple helix repeat-containing protein 1 (CTHRC1), lysyl oxidase (LOX), transforming growth factor beta induced (TGFBI), cadherin 11 (CDH11). (**C**) Western blot analysis of the abundance of CTHRC1 in CM from bone marrow-derived (BMSC) and adipose-derived (ASC) equine MSCs, isolated from donors of different age (*n* = 6). Heatmap bar shows the abundance of CTHRC1 (LFQ intensity) measured in CM by mass spectrometry. Fibronectin (FBN) was included as loading control (i). Density of CTHRC1 protein bands relative to FBN were measured with ImageJ and plotted against LFQ intensity of CTHRC1 in CM from the same MSC samples (ii). (**D**) Scatterplot showing enzymatic activity of LOX in MSC CM (*n* = 27), measured with a commercial fluorescence assay. The y-axis shows the difference in fluorescence intensity (RFU, relative fluorescence units), normalized to total protein concentration, between 30 minutes and 10 minutes incubation time (delta RFU). (**E**) Scatterplot showing change in MCP-1 concentration in MSC CM in relation to donor age. Dots represent biological replicates, lines the mean protein abundance predicted by GLM, and shades 95% CI.

Time course clustering using the MFuzz approach, with age as a time factor, identified 16 clusters of coexpressed proteins (Fig. 4A, Supplementary Material 5). However, protein abundance trajectories in those clusters did not show correspondence with donor age. Similarly, dynamic time warping (DTW) with 8 clusters did not identify a clear trend in protein cluster abundance related to donor age (Supplementary Fig. S2C, Supplementary Material 6). Varying the clusters for Mfuzz and DTWclust did not affect the main observation of lack of correspondence between clusters and age trajectory.

Analysis of individual secreted proteins with GLM detected effect of donor age in 4 proteins (*P* < .05): CTHRC1, cadherin 11 (CDH11), lysyl oxidase (LOX), and transforming growth factor-beta-induced protein ig-h3 (TGFBI). However, following correction for multiple comparisons, only in CTHRC1 the *q*-value associated with the effect of donor age met the threshold needed to reject the null hypothesis (*q* = 0.04) (Fig. 4B, Supplementary Material 4.). The extracellular proteins, CTHRC1, LOX, and TGFBI, have been all assigned a high rank in cluster 3 identified by NMF (Supplementary Material 3) while CTHRC1 and TGFBI were assigned to the same DTW cluster (Supplementary Material 5). This evidence suggests that CTHRC1, TGFBI, and LOX may be coexpressed and show the same direction of abundance change (decline) related to cell donor age.

We validated the abundance change in 2 of these proteins, CTHRC1 and LOX, using orthogonal techniques. Western blot analysis of CTHRC1 showed that relative abundance corresponded with quantitative values from the LFQ LC-MS/MS proteomics approach (Fig. 4C). Analysis of LOX activity in MSC CM showed a significant effect of time-by-age interaction term (*b* = −0.05, *P*-value < .001), suggesting that the increase of fluorescence over time that reflects LOX enzymatic activity in MSC CM decreased with donor age (Fig. 4D). These data demonstrate that donor age is most likely not driving global changes in MSC CM proteome. However, it does negatively affect secretion of several proteins relevant to cell migration, cell-ECM interaction and collagen synthesis.

### Donor Age Is Associated with Increased Secretion of the SASP Marker MCP1 by Equine MSCs

The results of ELISA provide evidence of an association between MCP-1 concentration in MSC CM and donor age (*b* = 0.08, *P* = .04, Fig. 4E). The results do not show any associations between the donor sex or tissue source and MCP-1 concentration.

### Limited Evidence of the Effect of Donor Sex on Protein Abundance in Equine MSC CM

**Figure 5.**
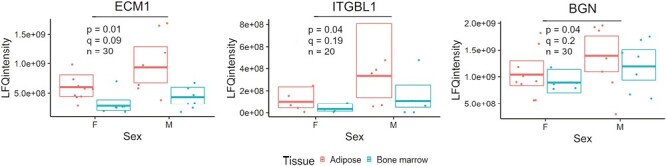
Effect of donor sex on abundance in equine mesenchymal stromal cell (MSC) conditioned media (CM). Dots represent biological replicates, boxes, and horizontal lines represent interquartile range and median predicted by generalized linear model (GLM). Number of data points obtained from different biological replicates included in each model is shown on the graphs. Colors designate tissue types used for MSC isolation. The proteins where the effect of donor sex on abundance was associated with *P*-value < .05 and *q*-value < 0.2 are shown: biglycan (BGN), extracellular matrix protein 1 (ECM1), and integrin subunit beta like 1 (ITGBL1).

Results of GLM analysis suggested that abundance of 3 proteins is higher in CM obtained from MSCs derived from male than female donors; however, following FDR correction for multiple comparisons, none of these effects met the threshold of *q* < <0.05 (Fig. 5, Supplementary Material 4). In order to reject null hypotheses related to the effect of donor sex on the abundance of these proteins, the significance threshold would need to be set at *q* < 0.2.

## Discussion

To our knowledge, this is the first study in any species reporting statistical analysis of a comprehensive protein profile of MSC CM obtained from donors of different age. Mass spectrometry was previously used to analyze the protein composition of ECM synthesized in vitro by human BMSCs from young and old donors.^59^ However, this study did not test for systematic differences in protein abundance but focused on proteins identified uniquely in either of the age groups. Other studies testing the effect of ageing on MSC CM all used the in vitro models of ageing through long-term culture, that are characterized by decreased cell proliferation capacity.^33-37^ Although replicative senescence reflects only one of the aspects of cellular ageing, increasing concentrations of MCP-1 in CM was observed both in MSCs from older horses and late passage human BMSCs.^33-35^ In vitro studies showed that the abundance of most CM proteins remains unchanged with progressive cell passaging^36,37^; however, proteins upregulated in late passage BMSCs had a senescence-inducing effect on early passage cells.^36^ Our results likewise suggests that the abundance of most proteins in equine MSC CM is unaffected by donor age, yet the age-related decrease in CTHRC1 and LOX may have functional implications, due to the role of these proteins in regulation of ECM remodeling.

Proteins, whose abundance was identified here as age-dependent, participate in regulating synthesis of collagen and elastin, the main structural components of ECM. CTHRC1 interacts with the TGF-β/Smad signaling pathway^60^ that controls activation of profibrotic genes. Expression of CTHRC1 has been linked with fibroblasts activation following injury in heart, arterial wall, and skin tissue, and with promotion of tissue healing through scar formation.^60-62^ LOX is an enzyme mediating post-translational modification (cross-linking) of collagen and elastin that leads to stabilization of fiber structure, affecting biomechanical properties of the ECM.^63^Age-related decline in the levels of CTHRC1 in equine MSC CM is in contrast with a human study which identified CTHRC1 only in ECM synthesized by BMSCs from old, but not young, donors.^59^ That discrepancy in the results could have been related to differences in donor species and experimental design, as well as limitations of data-dependent acquisition LC-MS/MS proteomics in detecting moderate- and low-abundant proteins.^64^

The effects seen here of tissue source on equine MSC CM protein content is in line with previous reports,^30-32^ notably, upregulation of CTHRC1 in BMSCs^30^ and clusterin (CLU) and decorin (DCN) in ASCs,^31^ which have previously been shown in human MSCs. In addition, our study showed higher variability in ASC secretome composition compared with BMSCs. The ASCs used in this study were isolated from horses undergoing gastrointestinal surgery, while the BMSCs were collected to generate autologous therapy for musculoskeletal disease. Although ASCs were collected only from cases without severe systemic symptoms, the health status of these horses would vary more than that of the BMSC donors. In the absence of any clear associations between known donor characteristics and NMF clusters, this could be considered as one of the potential factors driving variability in the ASC secretome. This finding puts into perspective the previously proposed concept of using fat tissue from laparotomy to isolate ASCs for allogenic therapeutic use.^28,65^

Interestingly, 6 MSC CM proteins that showed kinetic behavior of a classically secreted protein in the SIDLS experiment were annotated to intracellular and membrane compartments by GO CC terms. However, a review of the literature showed that all of these proteins were previously identified in a secretory form: destrin (DSTN), tripeptidyl-peptidase 1 (TPP1), and amyloid beta protein (APP) in MSC-derived extracellular vesicles (EVs)^66^; cadherin 11 (CDH11) in tumor-derived EVs,^67^ cathepsin V in secretory lysosomes,^68^ and tyrosyl-tRNA synthetase (YARS1) as a soluble cytokine.^69^ This demonstrates the value of dynamic labeling in studying cell secretomes, and especially proteins released by unconventional secretory routes.

The main limitation of this study is lack of flow cytometry characterization of MSC surface markers. Although evidence from gene expression and mass spectrometry analysis showed bulk expression of MSC markers and lack of expression of hematopoietic markers, it does not provide information about cell type proportions across the samples. This could have been a potential confounder in protein abundance analysis; therefore, future studies in MSC secretome variability should include flow cytometry characterization. To increase the likelihood that our results will be functionally relevant, we focused on analysis of proteins secreted by MSCs. However, conclusions about the functional implications of the present findings are limited to the previous knowledge about the role of those proteins in extracellular niche regulation. Further studies are needed to determine the effect of secretome composition on cell function using relevant in vitro models. Nevertheless, the results presented here may contribute to our understanding of the variability of the biological effects of MSCs through linking protein secretion to fundamental characteristics of a cell donor.

## Conclusions

Tissue type and donor age contribute to the heterogeneity in protein composition of MSC CM and should be taken into consideration when choosing the source of MSCs used for therapeutic applications.

## Supplementary Material

sxad060_suppl_Supplementary_Materials

## Data Availability

Raw data from mass spectrometry proteomic analysis and MaxQuant search output files are available in the ProteomeXchange Consortium via the Proteomics Identification Database (PXD039652). Code used for mass spectrometry data analysis is available at github.com/CBFLivUni/Turlo_MSC_secretome_proteomics and github.com/aturlo/MSC_secretome_proteomics. Other data that support the findings of this study are presented within the article and its figures.
