# Supplementary material for: Mesenchymal Stromal Cell Secretome Is Affected by Tissue Source and Donor Age
Source: Stem Cells. 2023 Aug 17;41(11):1047–59. doi: 10.1093/stmcls/sxad060 (PMC10631804; doi:10.1093/stmcls/sxad060)
Supplement: sxad060_suppl_Supplementary_Materials [file sxad060_suppl_supplementary_materials.zip › sxad060_suppl_Supplementary_Legends.docx]

**Supplementary Files**

**Supplementary File 1 (pdf). Details of experimental methods and data analysis.**

**Supplementary File 2 (xlsx). List of proteins and their kinetic parameters determined by Stable Isotope Dynamic Labelling of Secretomes (SIDLS) in equine bone marrow-derived mesenchymal stromal cell conditioned media.**

**Supplementary File 3 (xlsx). List of clusters and protein ranks assigned based on non-negative matrix factorisation clustering of proteins identified in equine mesenchymal stem cell conditioned media.**

**Supplementary File 4 (xlsx). Results of generalised linear model analysis of the relationship between donor age, sex and tissue source and abundance of secreted proteins in equine mesenchymal stromal cell conditioned media.**

**Supplementary File 5 (xlsx). List of protein clusters assigned based on dynamic time warping clustering of proteins identified in equine mesenchymal stem cell conditioned media.**

**Supplementary File 6 (xlsx). List of protein clusters assigned based on MFuzz clustering of proteins identified in equine mesenchymal stem cell conditioned media.**

**Supplementary Figures**

**Supplementary Figure 1. PCA plots of label-free LC-MS dataset resulting from analysis of equine mesenchymal stromal cell conditioned media with different imputation methods and non-imputed.**

**Supplementary Figure 2. Relationship between equine mesenchymal stromal cell (MSC) donor age and clustering of conditioned media (CM) samples based on their proteomic profile.** A, Principal Component Analysis (PCA) of MSC CM by age after removal of the tissue factor. Colour corresponds with MSC donor age range divided in 5-year intervals. B, Non-negative matrix factor (NMF) clustering rank validation to assign cluster number. C, Results of DTW time course clustering of MSC CM proteins.
