# Supplementary figures and images for: Mesenchymal Stromal Cell Secretome Is Affected by Tissue Source and Donor Age
Source: Stem Cells. 2023 Aug 17;41(11):1047–59. doi: 10.1093/stmcls/sxad060 (PMC10631804; doi:10.1093/stmcls/sxad060)

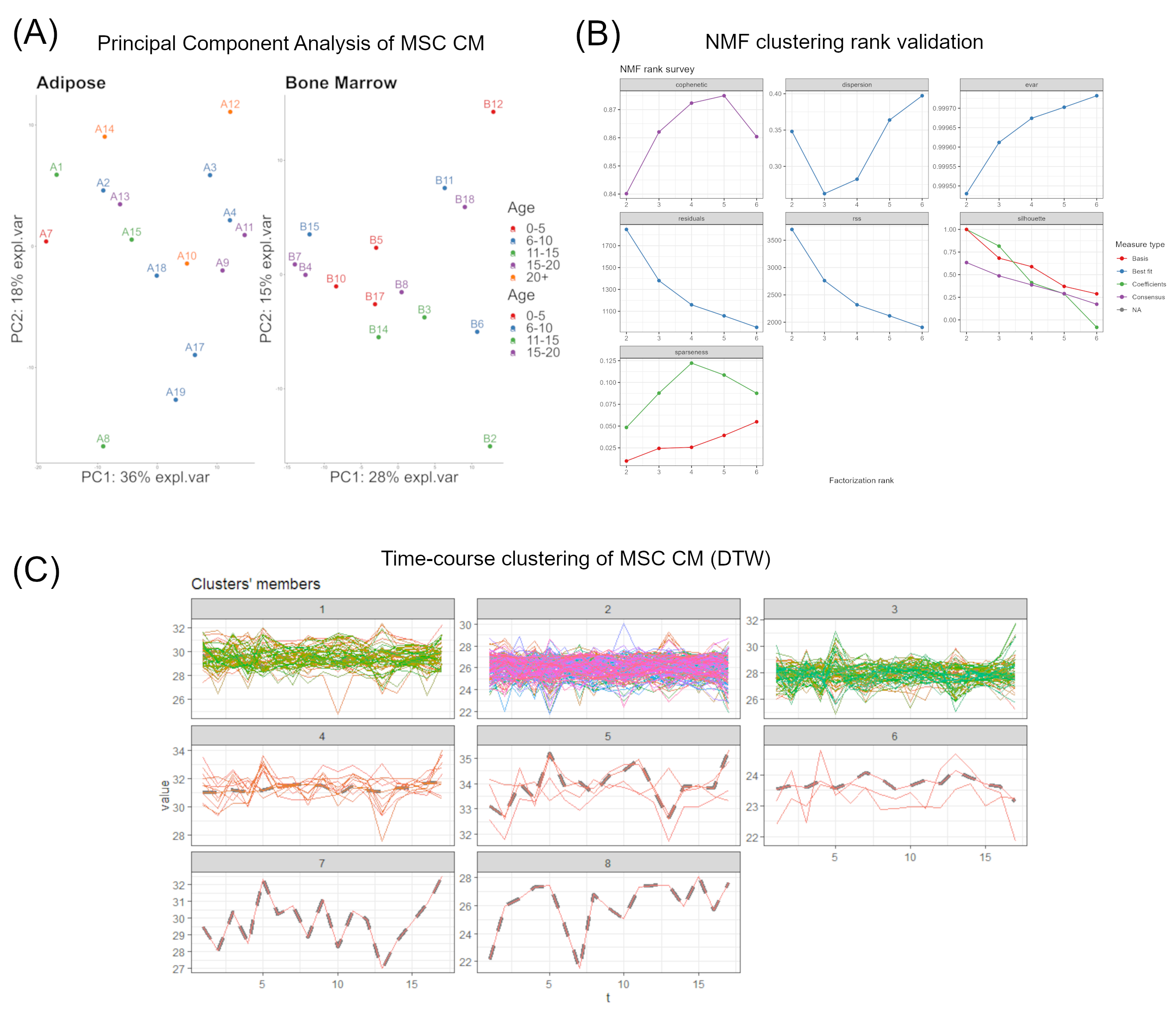

Supplement: sxad060_suppl_Supplementary_Materials [file sxad060_suppl_supplementary_materials.zip › sxad060_suppl_Supplementary_Figure_S2.tiff]

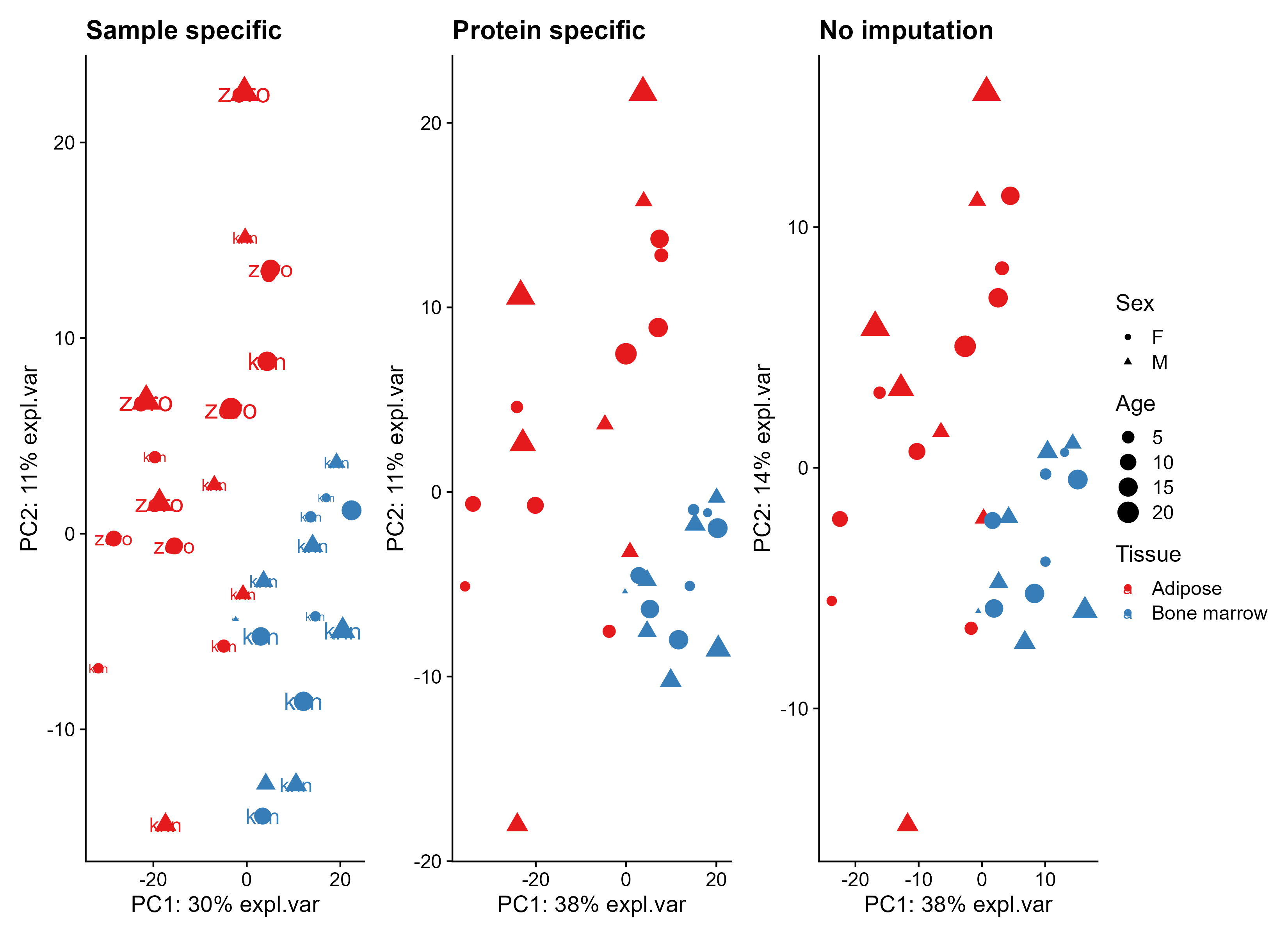

Supplement: sxad060_suppl_Supplementary_Materials [file sxad060_suppl_supplementary_materials.zip › sxad060_suppl_Supplementary_Figure_S1.tiff]
